# Taking a Closer Look: An Exploratory Analysis of Successful and Unsuccessful Strategy Use in Complex Problems

**DOI:** 10.3389/fpsyg.2019.00777

**Published:** 2019-05-07

**Authors:** Matthias Stadler, Frank Fischer, Samuel Greiff

**Affiliations:** ^1^Psychologie und Pädagogik, Ludwig-Maximilians-University Munich, Munich, Germany; ^2^Computer Based Assessment, University of Luxembourg, Luxembourg, Luxembourg City, Luxembourg

**Keywords:** log-file, problem-solving, n-grams, process data, educational assessment

## Abstract

Influencing students’ educational achievements first requires understanding the underlying processes that lead to variation in students’ performance. Researchers are therefore increasingly interested in analyzing the differences in behavior displayed in educational assessments rather than merely assessing their outcomes. Such analyses provide valuable information on the differences between successful and unsuccessful students and help to design appropriate interventions. Complex problem-solving (CPS) tasks have proven to provide particularly rich process data as they allow for a multitude of behaviors several of which can lead to a successful performance. So far, this data has often been analyzed on a rather aggregated level looking at an average number of actions or predefined strategies with only a few articles investigating the specific actions performed. In this paper, we report the results of an exploratory analysis of CPS log-files that is aimed at distinguishing between students that applied the correct strategy to a problem but failed to solve it and those applying the strategy successfully. In that, the sequence of behavior displayed is reduced to interpretable parts (n-grams) that allow searching for meaningful differences between the two groups of students. This level of analysis allows finding previously undefined or unknown patterns within the data and increases our understanding of the processes underlying successful problem-solving behavior even further.

## Introduction

The advent of computers to psychological and educational assessment has made it possible to analyze behavioral processes and sequences of actions through information captured in computer-generated log-files (records of all actions taken while working on a computerized assessment; Bunderson et al., 1989). Researchers are no longer limited to measuring the final outcome of an assessment (e.g., solved vs. not solved) but can also investigate the steps and actions resulting in the specific outcome through analyzes of test-taking behaviors. In other words, analyzing log-files allows researchers to make inferences about the latent cognitive processes involved in solving tasks from overt behavior (Greiff et al., 2015b). Log-files may, for example, inform researchers of specific mistakes made while working on a problem that may be indicative of a misunderstanding of the problem at hand (Ifenthaler et al., 2012). Identifying specific test-taking behaviors that lead to successful and unsuccessful performance has proven to be a treasure chest for the improvement of interventions and teaching enabling the differentiation of instructions and scaffolding and providing students with avenues for learning individually tailored to their needs.

A field that has made much use of log-file analysis in the last years is the field of complex problem-solving (CPS; e.g., Goldhammer et al., 2014; Greiff et al., 2016). Analyzing students’ behavior through log-files, it was shown that the application of the vary-one-thing-at-a-time strategy (VOTAT; Tschirgi, 1980), also referred to as “control of variables strategy” (Chen and Klahr, 1999), could explain a great deal of students’ performance in solving complex problems (Greiff et al., 2015b). Others noted, however, that simply identifying those students that applied the VOTAT strategy is not sufficient to fully explain why some students successfully solve a task whereas others do not (Kuhn and Dean, 2005). There must be other differences in metastrategic behavior that distinguish students that apply the VOTAT strategy and successfully solve a problem and those students that apply the strategy but fail. The aim of this paper is to use data mining techniques to analyze CPS log-files to find differences in behavior that indicate successful and unsuccessful behavior beyond the already established strategies.

## Log-Files in Complex Problem-Solving Tasks

Traces of behavior have been gathered in psychology studies since the 1930s (Skinner, 1938). Today, modern computer-based applications of psychological assessment make it very easy to capture a variety of interaction behaviors and save them to log files for later analysis. These interaction data have been referred to virtually synonymously as “log-file data” (Arroyo and Woolf, 2005), “discrete action protocols” (Fu, 2001), or “process data” (Zoanetti, 2010), only listing the most common names. Behavioral log-files are indicators of human behavior as observed by automatic sensors that capture and record actions displayed while interacting with the assessment. They may include behavior as diverse as rich audio and video recordings or low-level keystrokes.

Complex tasks, allowing for multiple behaviors that lead to a correct solution, produce valuable log-files with sufficient variation among participants for a meaningful interpretation. The study of how individuals engage with such complex tasks is therefore synonymous with problem-solving (Vista et al., 2016). Exploration of the processes employed in problem-solving or in engaging with complex tasks can provide information about the cognitive skills that underlie successful resolution of the problems or tasks (O’Neil et al., 2003; Griffin and Care, 2015). Indicators of these cognitive skills can be deduced from behaviors, which are captured in the form of attempted or completed processes in problem-solving tasks.

Problem-solving tasks that are particularly rich in log-file data are CPS tasks. Throughout this paper, CPS is understood as “(…) the successful interaction with task environments that are dynamic (i.e., change as a function of the user’s interventions and/or as a function of time) and in which some, if not all, of the environment’s regularities, can only be revealed by successful exploration and integration of the information gained in that process” (Buchner in Frensch and Funke, 1995, p. 14). CPS tasks thus differ from static problem-solving tasks in that they require active interaction between the problem solver and the problem resulting in very meaningful log-file data (Greiff et al., 2015a).

## Analyzing Log-File Data

### *A priori* Established Sequences of Behavior

Log-file data can be analyzed in two different ways: Based on *a priori* established sequences of behaviors (top–down) or bottom–up in an exploratory analysis that searches for patterns within the behavior displayed (Vista et al., 2016). Regarding CPS, various studies provided valuable findings by searching test-taking behavior for instances of specific, theoretically defined exploration strategies (e.g., Kröner et al., 2005; Wüstenberg et al., 2014). One of the strategies investigated most often in CPS research is the application of the VOTAT; Tschirgi (1980), also referred to as “control of variables strategy” (Chen and Klahr, 1999). When applying the VOTAT strategy, all variables of a problem are manipulated individually while the remaining variables are held constant to determine the effect of the varied independent variables on the dependent outcomes. VOTAT thus describes the principle of isolated variation of variables, which is the core component of scientific experimentation (Kuhn and Dean, 2005) and has been the almost exclusive focus of psychologists investigating the development of scientific reasoning (Zimmerman, 2000).

Empirically, multiple studies (e.g., Kröner et al., 2005; Wüstenberg et al., 2012, 2014) showed that application of VOTAT is strongly related to CPS performance (see also Funke, 2010). Most prominently, Greiff et al. (2015b) demonstrated the usefulness of the VOTAT strategy to explain performance differences within a problem-solving task that was part of the 2012 cycle of the Programme for International Student Assessment (PISA), one of the most widely recognized educational large-scale assessments (Turner and Adams, 2007). Their analysis of the *Climate Control* task showed, that applying the VOTAT strategy was strongly related to overall performance. This relation was observed both on the individual level and on the country level. However, not all students applying the VOTAT strategy solved the task leading researchers to search for other behaviors separating successful and unsuccessful problem-solvers (Kuhn and Dean, 2005).

As the empirical approach of searching for predefined behavioral patterns cannot explain why some students fail to solve tasks even though they apparently apply the correct strategy, it is necessary to take a closer look and conduct exploratory analyses searching for differences within the behaviors of students that apply the correct strategy and succeed and those that apply the correct strategy but fail, which is what we will attempt in this paper.

### Exploratory Approaches

Especially if long sequences of behavior need to be analyzed, exploratory approaches provide a helpful description of the underlying patterns. An approach proposed to explorative search for repetitive patterns within long sequences is the n-gram method (Damashek, 1995). The n-gram method summarizes a long string of entries (e.g., letters in words or separate instances of behaviors) as sequences of n consecutive elements. While this method was originally developed to classify and mine text data (Damashek, 1995), data scientists quickly noticed that it was also useful to classify behavior (mostly in the domain of web data mining; Mobasher, 2007). In this paper, we will use the n-gram approach to exploratively search for differences in behavior displayed by students that applied the VOTAT strategy to solve a complex problem and succeeded in solving it and those who applied the VOTAT strategy but failed to solve the problem.

To illustrate the n-gram approach take a problem that only allows for two different behaviors (A and B). A potential string of behaviors for the problem-solving process of a problem solver could look like this:

AABBBABABBBABBBAAABBABBBA

Table 1 illustrates how this sequence could be summarized by n-grams of the lengths *n* = 2 (bigrams), *n* = 3 (trigrams), and *n* = 4 (four-grams), each representing an increasingly more complex but less frequently appearing set of consecutive actions. In that way, the behavior of each problem solver could be described based on a set of sequences, which could then be used to either classify problem-solvers or predict future behavior (Liu and Kešelj, 2007). Due to this flexibility, n-grams form the basis of many data mining techniques (Borges and Levene, 2000).

**Table 1:** Example of n-grams of different length with respective frequencies.

*n* = 2		*n* = 3		*n* = 4	

Sequence	Frequency	Sequence	Frequency	Sequence	Frequency
AA	2	AAA	1	AAAA	0
AB	6	AAB	2	AAAB	1
BA	6	ABB	5	AABA	0
BB	5	ABA	1	AABB	2
		BAA	1	ABAA	0
		BAB	3	ABAB	1
		BBA	5	ABBA	1
		BBB	4	ABBB	4
				BAAA	1
				BAAB	0
				BABA	1
				BABB	3
				BBAA	1
				BBAB	3
				BBBA	4
				BBBB	0

## This Study

The aim of this study is to use exploratory educational data mining techniques in explaining CPS behavior. We go beyond the already established VOTAT strategy, exploring differences in behavior between students that applied the VOTAT strategy to a complex problem and successfully solved it and those that applied the strategy but failed to solve the complex problem. To analyze students’ behavior, we chose the n-gram approach (Damashek, 1995) introduced above to classify students that applied the VOTAT strategy into successful and unsuccessful problem-solvers based on their behavior. Applying the n-gram approach, we summarize the participating students’ behavior while solving the complex problem into a set of short sequences that can be used to find behaviors that are indicative of whether a student that applied the VOTAT strategy will also solve the complex problem. Next to presenting the empirical example, we will illustrate the methodological steps necessary to apply the n-gram approach to log-file data of CPS behavior.

## Empirical Example

### Sample

For the empirical example, we relied on a large sample (*N* = 1399) of students attending the ninth grade in a Finish municipality. The data were drawn from the Vantaa panel study for the development of learning to learn competencies in basic education. This panel is sampled to be representative for the Finish population based on several demographic and socioeconomic indicators (see Vainikainen, 2014 for more information) and the findings gained are likely to be generalizable to other samples. The mean age of the students at the time of data collection was 15.8 years (*SD* = 0.43). 48% of the students were girls and 50% boys (2% missing information). The data used for this study can be found in an anonymized form on the open science framework repository created for this paper^1^. The research design and the scales were approved by the local Education Department. The same scales and design have been used also in national educational evaluations commissioned by the Ministry of Education and Culture, and by the Finnish National Board of Education, based on the Basic Education Act (1999). The measures and design have been approved, in relation with another study, also by the Ethical Committee of the Finnish National Institute for Health and Welfare. Both the students and their parents were asked to provide their informed consent in writing.

### Task

Over the course of the assessment, students solved multiple CPS tasks based on the MicroDYN approach (Greiff et al., 2015a). The MicroDYN approach is based on linear structural equations (Funke, 2001) in which (in this study) three input variables were related to three output variables (see specific example below). The underlying relations were opaque to students at the onset of the task and needed to be determined by applying adequate strategies (i.e., the VOTAT strategy) to acquire knowledge about the problems’ structure and to apply that knowledge to achieve certain goals.

**FIGURE 1:**
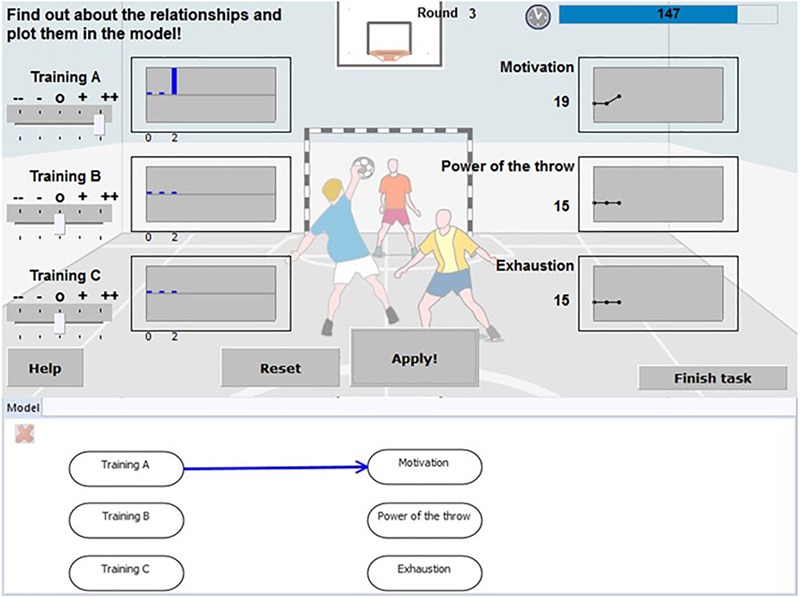
Scenario (top) and model (bottom) of the “Handball training” task.

The example task used for this paper was the item “Handball training,” which is illustrated in Figure 1. It illustrates problems based on the MicroDYN approach very well and is of sufficient difficulty to allow for variation in both behavior and successful solutions (Stadler et al., 2016). In this task’s scenario, participants take over the role of the coach of a handball team trying to figure out how different types of training (labeled Training A, Training B, and Training C; left part of Figure 1) influence certain attributes of the players (i.e., Motivation, Power of the throw, Exhaustion; right part of Figure 1). The best strategy to solve such tasks based on the MicroDYN approach is to apply the VOTAT strategy; that is, to manipulate each variable individually (e.g., to put Training C on “++”), while keeping all other input variables constant, and to click on “apply” (in the center of Figure 1). The resulting changes in the outcome variables indicate the relations between the input and the output variables. After working on the scenario, the resulting knowledge (i.e., the relation between the three training strategies and the three outcomes needed to be plotted in the model underneath the task; see the lower part of Figure 1).

### Extraction and Scoring of Log-Files

The task was implemented in the CBA item builder, a generic assessment platform, which has been designed to meet these requirements (for an overview see Rölke, 2012). This tool is provided by the German Institute for International Educational Research (DIPF) that organizes the development of the software and collects and coordinates new requirements. It allows users without programming experience to develop and deploy computer-based assessment tasks using a graphical user interface. After testing, log-files containing the response data can be downloaded in an XML format^2^ from the test computer or server for further analysis. A detailed description of the embedding and scoring of CPS tasks implemented in the CBA item builder can be found in Greiff et al. (2013). An exemplary XML file can be found on the open science framework repository see text footenote^1^.

For this study, we used two different scripts to extract the data from the log-files. To extract students’ scores, time on task, and the use of VOTAT, we used an SPSS script already used in various previous studies (e.g., Greiff et al., 2013). To score the application of the VOTAT strategy, log-files of students’ behavior were analyzed. Full credit was given if participants manipulated each input variable at least once while keeping all other variables constant; otherwise, no credit was assigned (Wüstenberg et al., 2014). For the explorative n-gram analyses the complete string of behaviors was extracted by a customized python script^3^, using the built-in xml.etree.ElementTree package.

Table 2 shows some exemplary data from a log file of the “Handball training” task. Students could make and apply changes to the input variables (i.e., the rounds in the assessment). One round was recorded every time the “Apply” button was pressed applying changes from none to all of the input variables (i.e., working on the scenario, “S”) to the output variables. In the example, the participant applied the VOTAT strategy in manipulating each variable individually. The nature of the XML files does, however, not allow discerning the order in which variables were manipulated within each round. Changes in the model were recorded every time a line was drawn or removed between an input and an output variable to plot findings (i.e., working on the model, “M”). Our python script extracted the string of behavior as a vector of “M” or “S” for each participant allowing for an easy interpretation. In the example, the extracted string would be “SSS” as all three recorded behaviors were changes in the scenario. In addition, we extracted the total number of behaviors (length of the vectors) and the time spent working on the task (without reading the problem description, which was presented separately from the actual problem scenario). Use of the “reset” and “help” button was ignored as these do not provide any additional information on the solution process and were used by a marginal number of participants (N_reset_ = 53; N_help_ = 19). The python script can be found on the open science framework repository see text footnote^1^.

**Table 2:** Example data from a log file of the “Handball training” task (adapted from Greiff et al., 2013).

General information		Input variables			Output variables		
Timestamp	Button pressed	Training A	Training B	Training C	Motivation	Power	Exhaustion
15:13:21	Apply	0	1	0	17	15	15
15:13:23	Apply	0	0	1	17	17	15
15:13:26	Apply	1	0	0	17	19	17

### Statistical Analysis

In order to find behavioral differences between students that applied the VOTAT strategy and successfully solved the complex problem and those students that applied the VOTAT strategy but failed, we first identified the respective students, assigning a dummy coded variable. This variable separated successful and unsuccessful students that applied the VOTAT strategy and assigned missing values to all students that did not apply the VOTAT strategy.

To find the sequences of behaviors that led to success or failure in the problem-solving process, we applied the chi-square feature selection model, which is frequently used in natural language processing or other data mining contexts (Oakes et al., 2001). Recent publications have demonstrated how to apply this approach to problem-solving data, though (He and von Davier, 2015, 2016). The chi-square feature selection model tests whether occurrence and non-occurrence of behaviors are independent for two groups. Under the null hypothesis, the behaviors would be equally likely for both groups. Based on the observed distribution of behaviors, a chi-square value can thus be computed to evaluate the departure from this null hypothesis. A problem with this approach is potentially over-interpreting the relevance of extremely common behaviors that have little or no discriminating power while under-estimating the relevance of rather infrequent behaviors. Moreover, the added relevance of a behavior is not linear. More occurrences of a behavior indicate higher importance, but not as much relative importance as an undamped count would suggest (Manning and Schütze, 2005). To solve this problem, a weight is assigned to the observed frequency of each sequence of behaviors based on the number of participants displaying the sequence of behavior, the sequence’s total frequency, and the total number of behaviors observed for a more detailed description of the chi-square feature selection model see (He and von Davier, 2016). The weight function for sequence of behavior *i* in total behavior *j* (1) was defined as:

(1) $$\mathrm{weigth}\left(i,j\right)=\;\{\begin{array}{c} \left[1+\log\left(f_{i,j}\right)\right]\log\left(\frac{N}{sf_{i}}\right)\;\;\;if\;f_{i,j}\geq 1\\ 0\;\;\;\;\;\;\;\;\;\;\;\;\;\;\;\;\;\;if\;\;f_{i,j}=0 \end{array}$$

where *N* is the total number of sequences, f is the sequence’s frequency and sf is the number of behaviors where the sequence i appears. The first clause applies to sequences occurring in the same behavior, whereas for sequences that do not appear (f*_i,j_* = 0), we use weight (*i,j*) = 0.

The scripts for all analyses can be found on the open science framework repository see text footnote^1^. Table 3 provides the raw and weighted frequencies for all sequences of behavior of students applying the VOTAT strategy.

**Table 3:** The raw and weighted frequency for all sequences of behavior.

Behavior sequence	Frequency of sequences	Frequency of actions	Weight	Freq. in correct		Freq. in incorrect	
				Raw	Wgt	Raw	Wgt
Bigrams							
MM	663	5063	0.01	4073	37.57	990	9.13
MS	159	221	2.08	166	345.36	55	114.43
SM	224	327	1.66	249	414.13	78	129.73
SS	81	226	3.07	156	478.76	70	214.83
Trigrams							
MMM	628	4275	0.12	3454	408.11	821	97.01
MMS	125	159	2.33	120	279.12	39	90.71
MSM	118	147	2.38	113	269.00	34	80.94
MSS	54	66	3.08	47	144.59	19	58.45
SMM	206	267	1.75	208	363.20	59	103.02
SMS	28	33	3.47	25	86.66	8	27.73
SSM	79	96	2.76	72	198.80	24	66.27
SSS	50	128	3.49	82	286.51	46	160.73
Four-grams							
MMMM	495	3574	0.59	2895	1698.67	679	398.41
MMMS	80	101	2.77	79	218.45	22	60.83
MMSM	90	103	2.62	80	209.51	23	60.23
MMSS	41	48	3.25	34	110.37	14	45.45
MSMM	97	114	2.56	90	230.19	24	61.39
MSMS	14	15	3.65	11	40.15	4	14.60
MSSM	22	23	3.50	17	59.46	6	20.99
MSSS	36	41	3.31	28	92.70	13	43.04
SMMM	171	210	1.96	167	327.60	43	84.35
SMMS	24	28	3.53	22	77.70	6	21.19
SMSM	20	21	3.54	16	56.57	5	17.68
SMSS	12	12	3.63	9	32.64	3	10.88
SSMM	67	76	2.87	60	172.40	16	45.97
SSMS	11	11	3.64	8	29.10	3	10.91
SSSM	50	57	3.10	40	123.96	17	52.68
SSSS	25	71	4.06	42	170.71	29	117.87

*Freq., frequency; Wgt, weight; S, working on the scenario; M, changing the model.*

## Results

As can be seen from Table 4, the task was relatively difficult with only 544 (38.9%) of the students solving the task correctly. Moreover, 666 (47.6%) of the students applied the VOTAT strategy. Applying the VOTAT strategy, generally, lead to a substantially higher likelihood of solving the problem (χ^2^ = 401.10; *df* = 1; *p* < 0.001). However, 143 (21.5%) of the students that applied the VOTAT strategy did not solve the problem.

**Table 4:** Distribution of students based on whether they solved the problem and applied the VOTAT strategy.

Applied the VOTATstrategy				
	No		Yes	Total
Solved the problem	No	712	143	855
	Yes	21	523	544
Total		733	666	1399

In the exploratory analysis, we attempt to understand this observation by finding behavioral differences among the students that applied the VOTAT strategy by using the n-gram approach. There was no significant difference between the absolute number of behaviors observed for either group of students [*t*(664) = 0.52; *p* = 0.601; *d* = 0.05] nor the time spent working on the task [*t*(664) = 0.27; *p* = 0.790; *d* = 0.03]. Table 5 displays the results of the chi-square feature selection model analyzing differences in likelihoods of specific n-grams for students that applied the VOTAT strategy and solved the problem and those that did not. Note that the possible behaviors were reduced to working on the scenario (S) and changing the model (M). N-grams with higher chi-square values are more discriminative between the two groups. Moreover, Table 5 indicates whether the n-grams were more typical of students that solved the problem or of those that did not.

**Table 5:** Summary of the chi-square feature selection model for bigrams, trigrams, and four-grams.

*n* = 2				*n* = 3				*n* = 4			
Sequence	χ^2^	*p*	Dir.	Sequence	χ^2^	Dir.	*p*	Sequence	χ^2^	Dir.	*p*
SS	31.98	<0.001	-	SSS	59.98	-	<0.001	SSSS	76.34	-	<0.001
MM	0.92	0.337	+	MMM	12.16	+	<0.001	MMMM	67.09	+	<0.001
MS	0.23	0.632	-	MSS	4.08	-	0.043	MSSS	7.21	-	0.007
SM	0.05	0.823	+	SMM	1.95	+	0.163	SMMM	5.43	+	0.020
				MSM	0.37	+	0.543	SSSM	5.30	-	0.021
				SSM	0.17	-	0.680	MMSS	3.64	-	0.056
				MMS	0.04	-	0.841	MSMM	2.70	+	0.100
				SMS	0.00	-	1.00	SSMM	2.01	+	0.156
								MMMS	1.52	+	0.218
								MMSM	0.87	+	0.351
								SMMS	0.69	+	0.406
								SSMS	0.36	-	0.549
								MSMS	0.32	-	0.572
								MSSM	0.27	-	0.603
								SMSS	0.03	-	0.862
								SMSM	0.01	+	0.920

*N-grams with higher chi-square values are more discriminative; Dir., Direction of the difference between groups. “+” represents behaviors that were more typical of students that solved the task “-“ represents behaviors that were more typical of students that did not solve the task; S, working on the scenario; M, changing the model.*

As can be seen from Table 5, the informational value of the n-grams increases with their length, while the general pattern does not change. The most discriminative sequence of behavior was consistently the one indicating working maximally long in the scenario (SS, SSS, and SSSS), which was always more typical of the students that did not solve the task. This was followed by the sequence of behavior indicating working maximally long in the model (only statistically significant for MMM and MMMM), which was always more typical of students solving the task. Generally, the sequences indicating repeated changes in the scenario were associated with failing to solve the problem (statistically significant for MSS, MSSS, and SSSM), whereas the sequences indicating repeated changes in the model were associated with solving the problem (statistically significant for SMMM). The discriminative value (high chi-square values) was highest for the sequences with the longest uninterrupted sequences of one specific behavior (M or S) and least for those that indicated frequent changes between working on the scenario and working on the model (e.g., SMSM).

## Discussion

The aim of this study was to use exploratory educational data mining techniques in explaining problem-solving behavior. We chose one of the most established types of CPS tasks (based on the MicroDYN approach; Greiff et al., 2015a), for which the optimal strategy is well known (i.e., the VOTAT strategy; Tschirgi, 1980). However, not all students applying the VOTAT strategy also solved the tasks correctly implying that simply observing whether or not the strategy was applied is not sufficient to understand why some students succeed in solving CPS tasks while others do not (Kuhn and Dean, 2005).

Describing the whole string of behaviors observed for each individual student as a set of n-grams of different length (Damashek, 1995) allowed us to exploratively search for differences in the behavior observed within those students that applied the VOTAT strategy and successfully solved the task and those that applied the strategy but still failed to solve the task. The empirical example illustrates that given enough complexity, there are substantial differences in the frequencies of observed n-grams between the two groups. Interpreting those differences, however, requires some understanding of the task and what it takes to solve it (Banovic et al., 2016).

Correctly applied, the VOTAT strategy requires problem-solvers to make only minimal changes in the scenario, register the effects and then immediately plot the findings in the model (Wüstenberg et al., 2012). Any deviations from this algorithm will increase the cognitive load (Sweller, 2011) on the problem-solver as important information (i.e., either changes made in the scenario or findings resulting from these changes) need to be stored in working memory (Sweller, 1988). Inspecting the differences in behaviors between students that applied the VOTAT strategy and successfully solved the task and those that applied the strategy but still failed, the general pattern seemed to be that the students that solved the task spent fewer rounds continuously working on the scenario (e.g., SS, SSS, or SSSS) but more rounds working on the model (e.g., MMM and MMMM). Students that did not solve the task, thus, did not immediately plot their findings, thereby increasing their cognitive load and, in turn, the task’s difficulty (Kirschner, 2002). Our findings thus highlight the importance of metastrategic competencies that enable a person to not only apply the correct strategy to solve a problem but to make use of the information gained in the process. Metastrategic competencies encompass awareness, understanding, monitoring, and management of one’s strategic performance of many kinds of cognitive tasks (Kuhn and Pearsall, 1998). As becomes obvious from our analyses, students that did not solve the problems correctly either lacked understanding of the VOTAT strategy or were not able to manage their use of the strategy. Due to the exploratory nature of our analyses, our interpretations are *post hoc* though and should be corroborated by experimental studies.

There are other limitations to be considered. In focusing only on the students applying the VOTAT strategy we reduced our sample to *N* = 666, excluding almost half of the initial sample from our analyses. However, since the aim of our paper was to find behavior differences between students that applied the VOTAT strategy and successfully solved the problem and those students that applied the VOTAT strategy but failed, students that did not apply the VOTAT strategy at all were irrelevant to our analyses. Future studies should extend our analyses to explore differences in behavior across all students not selected by *a priori* defined strategies.

Moreover, coding of the log-files into changes in the scenario and changes in the model does not allow differentiating between different changes applied to the input variables within one round of changes to the scenario (e.g., manipulations of only one variable vs. manipulations of multiple variables). However, this simplification allows for a relatively straight-forward interpretation of the resulting n-grams. A more detailed coding of changes to the scenario, on the other hand, would lead to an exponentially higher number of potential behavior sequences most of which would most likely have very little information value due to their specificity. The potential variance in changes in the input variables between successful and unsuccessful students is further reduced by the fact that all participants included in our analyses applied the VOTAT strategy (i.e., manipulated all input variables at least once individually while keeping the others constant). Since manipulating all input variables individually once is sufficient to solve the task, all further manipulations, regardless of whether single or multiple variables, will result in unnecessary additional information increasing cognitive load. Testing these assumptions will, however, require additional information to be logged (for more on the completeness of log data see Kroehne and Goldhammer, 2018).

Finally, the n-gram approach showcased in this paper is not the only explorative educational data mining approach applicable to CPS log-files, of course. Other studies have applied analyses of the interaction of behavior displayed while solving tasks such as Network Analysis (Wooldridge et al., 2018), or included the temporal order of behaviors in their analyses by displaying them as complex directed networks (Vista et al., 2016). All of these approaches share the aim of understanding problem-solving behavior on a very detailed level and the difficulties that come with that aim. Most importantly, any increase in task specificity (e.g., longer n-grams) comes, necessarily, with a decrease in generalizability. In that, perfect understanding of students’ behavior in one task may be meaningless to understand performance in another task unless the structural similarities between these tasks are well understood and theoretically described. Future studies should, therefore, investigate the generalizability of behavior across different problem-solving tasks.

The findings show the potential benefit of applying explorative educational data mining approaches such as the n-gram approach in addition to searching for *a priori* defined strategies. Knowledge about how and why students that actually apply the correct strategy to solve a problem fail to actually solve it has implications for the instruction or training of CPS tasks. So far, interventions aimed at increasing CPS performance have relied on repeatedly confronting problem-solvers with problems of a similar nature (e.g., Kretzschmar and Süß, 2015). Training lead to an increase in performance and, in fact, also to an increase in strategic prowess (Lotz et al., 2017). However, no dedicated strategy training has been published to the best of our knowledge. Based on our findings, such a strategy training should consider to not only teach the VOTAT strategy but also metastrategic knowledge such as the handling of information gained through the application of VOTAT (Zohar and Peled, 2008).

## Conclusion

In summary, our paper showcased the n-gram approach on a CPS task. The detailed description of the data provided some indication toward behavioral differences within students that apply the correct strategy toward a problem and solve it as opposed to those that apply the correct strategy and fail. We hope that the paper will help other scholars in finding ways to analyze and interpret log-file data themselves. After all, the exploitation of this rich resource through dedicated analyses is still in its infancy and we believe that it is a treasure trove worth hunting for.

## Ethics Statement

Ethics approval was granted for the data collection (see Vainikainen, 2014).

## Author Contributions

All authors listed have made a substantial, direct and intellectual contribution to the work, and approved it for publication.

## Conflict of Interest Statement

SG is one of two authors of the commercially available COMPRO test that is based on the multiple complex systems approach and that employs the MicroDYN approach. However, for any research and educational purposes, a free version of MicroDYN tasks is available and he receives royalties for COMPRO. The remaining authors declare that the research was conducted in the absence of any commercial or financial relationships that could be construed as a potential conflict of interest.

## Footnotes

1 osf.io/jycku

2 http://www.w3.org/XML

3 www.python.org
